# The bra strap incision in the open Latarjet procedure

**DOI:** 10.1186/s13018-018-1006-8

**Published:** 2018-11-29

**Authors:** Alexandra Vlajkovic, Dominik C. Meyer, Marius Von Knoch, Samuel L. Schmid, Tobias Götschi, Florian Grubhofer

**Affiliations:** 1 Department of Orthopaedics, University of Zurich, Balgrist University Hospital, Forchstrasse 340, CH-8008 Zürich, Switzerland; 2 Department of Orthopaedics and Traumatology, Kreiskrankenhaus Osterholz, Am Krankenhaus 4, 27711 Osterholz-Scharmbeck, Germany; 3 Department of Orthopaedics and Traumatology, Hospital Brig, Überlandstrasse 14, 3900 Spitalzentrum Oberwallis, Brig, Switzerland

**Keywords:** Scar coverage, Deltopectoral approach, Bra strap incision, Scar cosmetics, Latarjet procedure, Shoulder instability

## Abstract

**Background:**

The anterior deltopectoral approach is the standard approach for performing the open Latarjet procedure. Through the use of a more medial and vertical skin incision, the scar can be cosmetically covered by the bra strap in women. We call this incision the bra strap incision.

The intention of this study was (1) to elaborate if the bra strap incision is considered beneficial by female patients, (2) to find reproducible landmarks to indicate how the bra strap incision has to be oriented, and (3) to evaluate preliminary clinical results of patients in whom the bra strap incision was used.

**Methods:**

In 18 patients with a mean follow-up of 21 (range, 12–31) months treated with an open Latarjet procedure through the bra strap incision, the clinical results (scar satisfaction, Constant and Murley score [CMS], and subjective shoulder value [SSV]) were retrospectively analyzed.

To assess the typical course of the bra strap, anatomical landmarks were assessed in 100 consecutive female patients as the distance from the bra strap center to (1) the tip of the coracoid process, (2) the superior end of the anterior axillary fold, and (3) the acromioclavicular joint.

**Results:**

All (18 of 18) patients stated that they would prefer the bra strap incision if the same procedure had to be performed on the opposite shoulder; 16 women were satisfied with the scar. The mean CMS was 83 (range 64–96) points and the mean SSV was 85 (range, 60–100) %.

The mean distances from the bra strap center to the acromioclavicular joint, coracoid tip, and axillary fold were 28 (range, 5–60) mm, 15 (range, 2–17) mm, and 30 (range, 2–55) mm.

No combination of distance measures and demographic variable revealed a linear relationship.

**Conclusion:**

This analysis shows that the bra strap incision appears to be highly welcomed by female patients and does not compromise the clinical outcome, when compared to previously published data. However, even though the typical location of the bra strap can be determined, the large variations in the distances make it more preferable to preoperatively mark the incision for optimal placement.

**Trial registration:**

The study is approved by the Ethical Committee Zurich. (Cantonal Ethical Committee number: ZH-Nr.2017–00891).

## Background

**Fig. 1:**
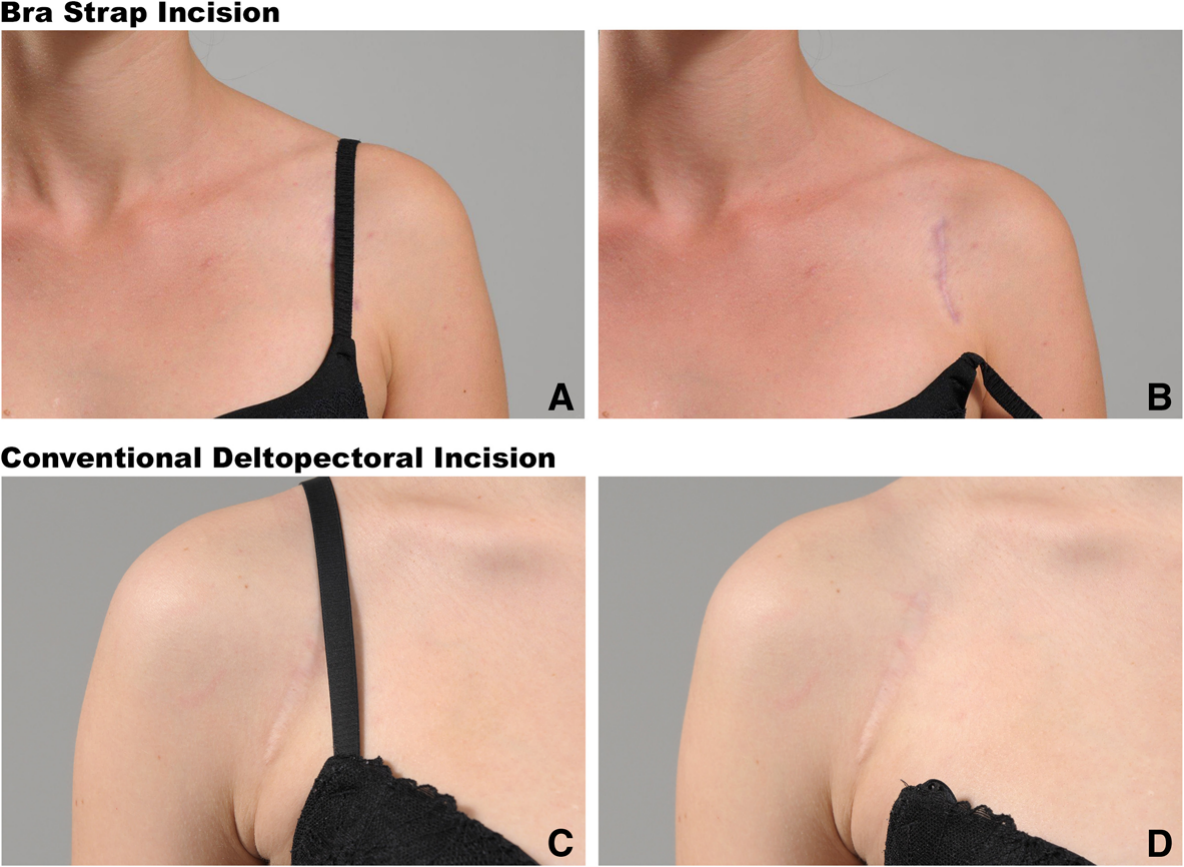
Comparison of the coverage of unfavorable scar formation with the bra strap incision (**a**,**b**) and conventional deltopectoral skin incision (**c**,**d**)

The deltopectoral approach to the shoulder, located on an interneural plane between the musculocutaneous and axillary nerve, is the standard approach for open anterior shoulder stabilization procedures [1–5]. The skin incision for this approach is usually drawn obliquely from the coracoid tip distally within the deltopectoral groove towards the proximal humerus [6]—see Fig. 1. The extension of the skin incision depends on the planned intervention. The open Latarjet procedure is one of the most common used stabilization techniques in case of recurrent anterior shoulder instability [7, 8]. The resulting scar lies in an exposed body region, which sometimes presents as an aesthetical issue especially in the young population who is often affected by anterior shoulder instability [9]. Leslie et al. described an axillary incision for the deltopectoral approach [10] to gain a cosmetic advantage because the incision is hidden in the axilla. For the Latarjet procedure, the axillary incision is located too far inferior as the coracoid process, which has to be addressed and mobilized, lies too far superiorly. Therefore, the axillary incision cannot be used for this population. It is known that scares that can be covered by clothes are desired by patients if the clinical result is not compromised [11]. By drawing the skin incision in a more vertical and medialized fashion, the resulting scar can be covered by the bra strap in women. We call this skin incision modification the “bra strap incision”. The positive feedback regarding the aesthetics of the coverable scar we got from women who undergone a Latarjet procedure through a bra strap leads us to perform this study. The purpose of this study was therefore (1) to evaluate whether the bra strap incision is considered beneficial by women, (2) to assess the preliminary clinical results of female patients who have already undergone the Latarjet procedure with the use of the bra strap incision, and (3) to elaborate constant anatomical shoulder landmarks with corresponding distances to the longitudinal bra strap center to enable prediction of the bra strap course.

## Methods

The study was performed at the Balgrist University Hospital, University of Zurich, Switzerland, after the Ethical Committee Zurich, Switzerland approved the study protocol (Cantonal Ethical Committee Nr.:2017-00891.

We retrospectively reviewed all female patients identified in our comprehensive database who underwent the Latarjet stabilization procedure with the use of a bra strap incision between January 2015 and January 2017. All female patients with a minimum follow-up of 12 months and a completed Constant and Murley Score [12] (CMS) were included for the analysis of the functional results.

In all patients, the bra strap course was marked preoperatively with a permanent marker so that the surgeon could set the bra strap incision intraoperatively. The Latarjet procedure was performed as described in previous studies from our institution [8, 13], according to the technique described by Walch [14], which is a modification of the original technique described by Latarjet [7].

The patient’s scar satisfaction was evaluated as the primary endpoint parameter via a questionnaire wherein the cosmesis of the scar could be judged as “very nice,” “nice,” “moderate,” or “not nice.” The women were asked if they would prefer the bra strap incision if the same procedure had to be performed on the opposite shoulder. Additionally, the patients were asked if they experienced any scar-specific discomfort or pain.

The shoulder function was assessed as the secondary endpoint with the CMS, the relative CMS [15], the subjective shoulder value [15], and the pain level, which was assessed within the CMS on a scale from 15 (no pain) to 1 (worst pain) points. To assess shoulder instability, the anterior apprehension [16] and anterior drawer tests [16] were performed in all 18 patients.

To predict the bra strap course on the skin of a female patient, three mean distances from shoulder anatomical landmarks to the longitudinal center of the bra strap were elaborated as the third outcome parameter. Therefore, we measured these distances in 100 female patients who were seen in our outpatient shoulder clinic between November and December 2017. All patients with no previous shoulder surgeries, no scars at the shoulder region, a cup size between A to D (European size), and who provided a signed consent form were included for the measurement study. For each cup size group (A to D), 20 women were consecutively recruited. The recruitment was stopped when each group reached 20 participants. As patient demographic characteristics, the actual cup size (European size A, B, C, or D), body weight in kilograms, and the body mass index (BMI) in kg/m^2^ were measured.

**Fig. 2:**
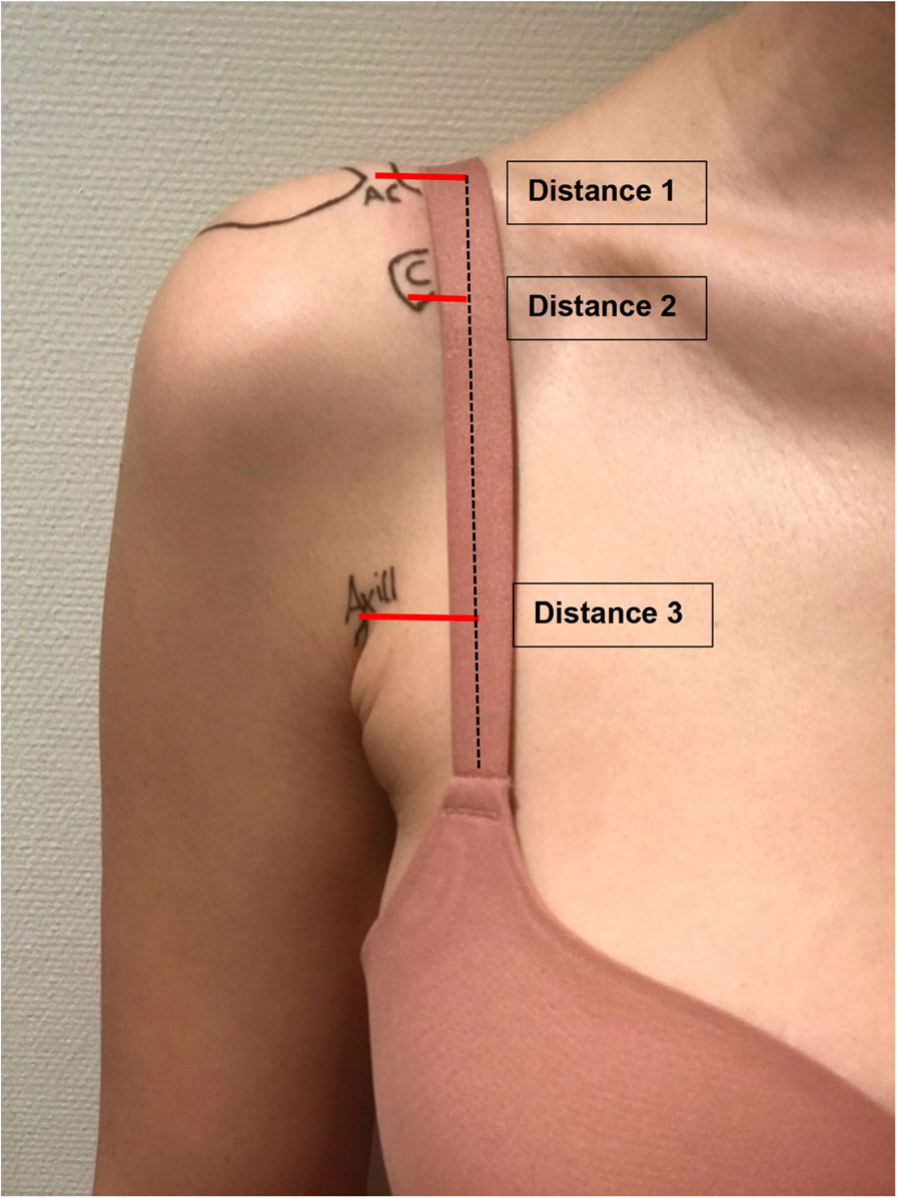
Distances from the anatomical landmark to the longitudinal center of the bra strap. Distance 1 = center of the acromioclavicular joint to the bra strap center. Distance 2 = tip of the coracoid process to the bra strap center. Distance 3 = top of the anterior axillary fold to the bra strap center

The shoulder landmarks were (1) the acromioclavicular joint line, (2) the tip of the coracoid process, and (3) the top of the anterior axillary fold. From these landmarks, the distance to the longitudinal center of the bra strap was measured in millimeters, see Fig. 2. The mean distance of each landmark to the longitudinal bra strap center with its coefficient of variation was evaluated as the tertiary endpoint. In an attempt to improve bra strap position prediction, the associations between distance measurements and demographic information (cup size, body weight, and BMI) were investigated as the secondary outcome parameters using ordinary least square linear regression including a constant in the equation. The *P* values were Bernoulli-corrected. *P* values < 0.05 were considered statistically significant.

The statistical analysis was performed by the co-author (TG) using SPSS (IBM SPSS Statistics for Windows, Version 22.0. Armonk, NY: IBM Corp.).

## Results

A total of 18 female patients who underwent anterior shoulder stabilization with the Latarjet procedure and using a bra strap incision were operated on in our institution between January 2015 and January 2017. Three trained shoulder surgeons from our institution performed the surgeries. The mean age of the patients was 29 (range 17–52) years, and the mean follow-up period was 21 (range, 12–31) months. All 18 patients were available for follow-up and were included in the study.

The 18 shoulders that were operated on comprised 11 right and 7 left shoulders; 10 were dominant and 8 were non-dominant. Among the 18 patients, 16 rated their scar as “very nice.” Two patients were moderately satisfied with the scar aesthetics; this was due to keloid formation in a 17-year-old patient and a broad scar formation in a 23-year-old patient. Both patients refused scar revision at the latest follow-up visit. All 18 patients stated that they would prefer the bra strap incision if the same procedure were to be performed on the opposite shoulder. The bra strap covered the scar in all patients in whom the bra strap course was marked preoperatively. No scar-specific complications were documented in all 18 cases, and none of the patients reported scar pain elicited by the pressure of the bra strap.

The mean absolute CMS was 83 (range 64–96) points, the relative CMS was 83 (range, 64–97) percent, the mean pain level was 14 (range, 8–15) points assessed using the CMS, and the mean subjective shoulder value (SSV) was 85 (range, 60–100) %.

All patients had a negative anterior apprehension and a negative anterior drawer test—Table 1.

**Table 1:** Demographics and functional outcome of 18 female patients after the Latarjet procedure using the “bra strap incision” technique, *n* = 18, *n* = 38

Follow up (months)	21 (12–31)
Age (years)	29 (17–52)
Right/left (shoulder)	11/7
Dominant/adominant (shoulder)	10/8
CMS points	83 (64–96)
RCS (%)	83 (64–97)
SSV (%)	85 (60–100)
VAS (pain) points	14 (8–15)
Negative anterior apprehension test	18
Negative anterior drawer test	18
Scar satisfaction	
“Very nice”	16
“Nice”	0
“Moderate”	2
“Not nice”	0
Bra strap incision desired	18

*n* number of patients, *CMS* Constant and Murley score, *RCS* Relative Constant Score, *SSV* subjective shoulder value, *VAS* visual analogue scale within the CMS ranging from 15 = no pain to 0 = worst pain

### Bra strap course measurements

The 100 consecutive women who participated in the bra strap course measurement had a mean age of 36 (range 17–55) years. The mean weight was 64 (range 50–95) kg, the mean BMI was 23 (range 18–35) kg/m^2^, and the mean height was 166 (range 152–176) cm.

**Fig. 3:**
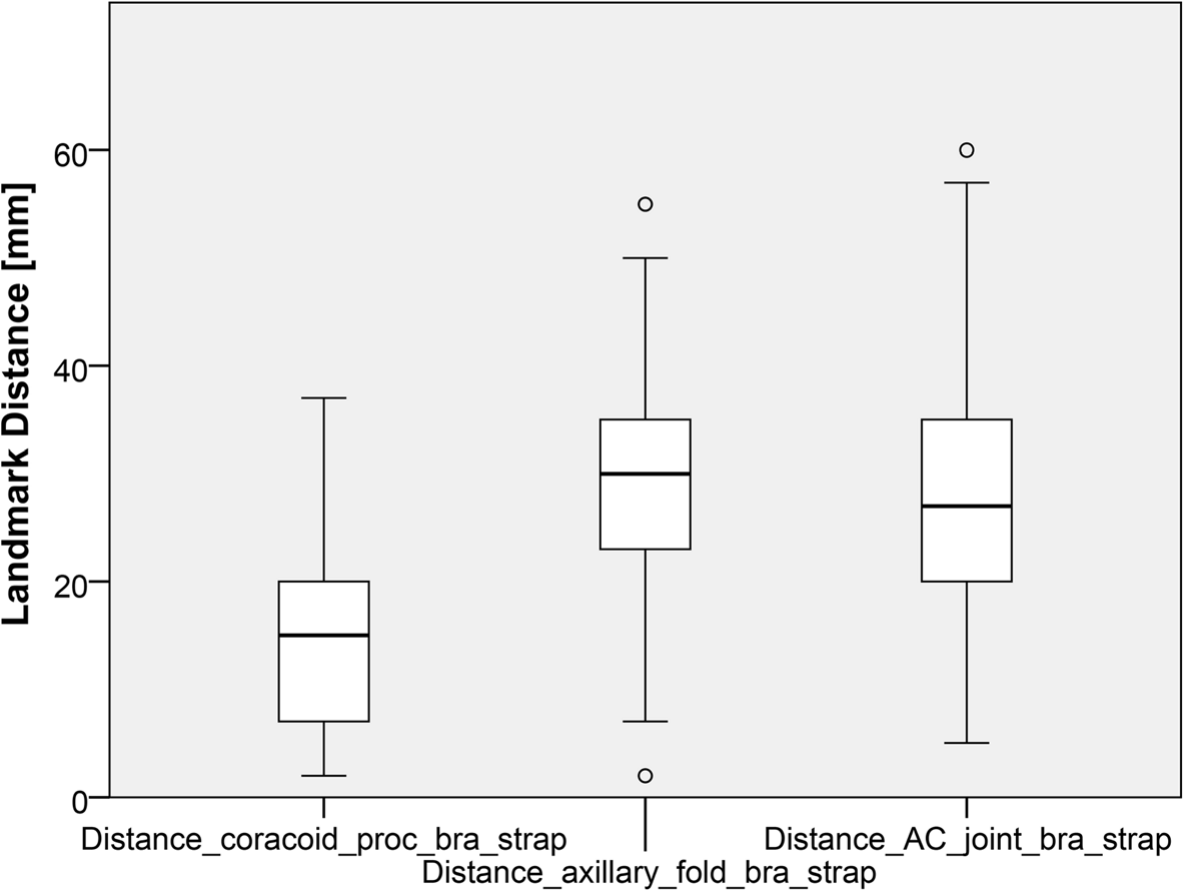
Boxplots of the median distances in mm from the three shoulder landmarks to the longitudinal center of the bra strap

The mean distances from the bra strap center to (1) the acromioclavicular joint line, (2) the tip of the coracoid process, and (3) the anterior axillary folds were 28 ± 10 mm, 15 ± 8 mm, and 30 ± 11 mm, respectively. However, the variability was considerable as outlined in Table 2 and Fig. 3.

**Table 2:** Demographics and distances from anatomical shoulder landmarks to the longitudinal center of the bra strap of 100 female patients

Age (years)	36 (range, 17–55)	
Weight (kg)	64 (range, 50–95)	
Body mass index (kg/m^2^)	23 (range, 18–38)	
Number of cup size (European size A, B, C, D)	Each group 20	
	Mean distances: landmark—bra strap	Coefficient of variation
AC joint (mm)	28 (range, 5–60, SD 10)	0.36
Coracoid process (mm)	15 (2–27, SD 8)	0.54
Axillary fold—bra strap (mm)	30 (2–55, SD11)	0.37

*AC* acromioclavicular joint, *SD* standard deviation

**Fig. 4:**
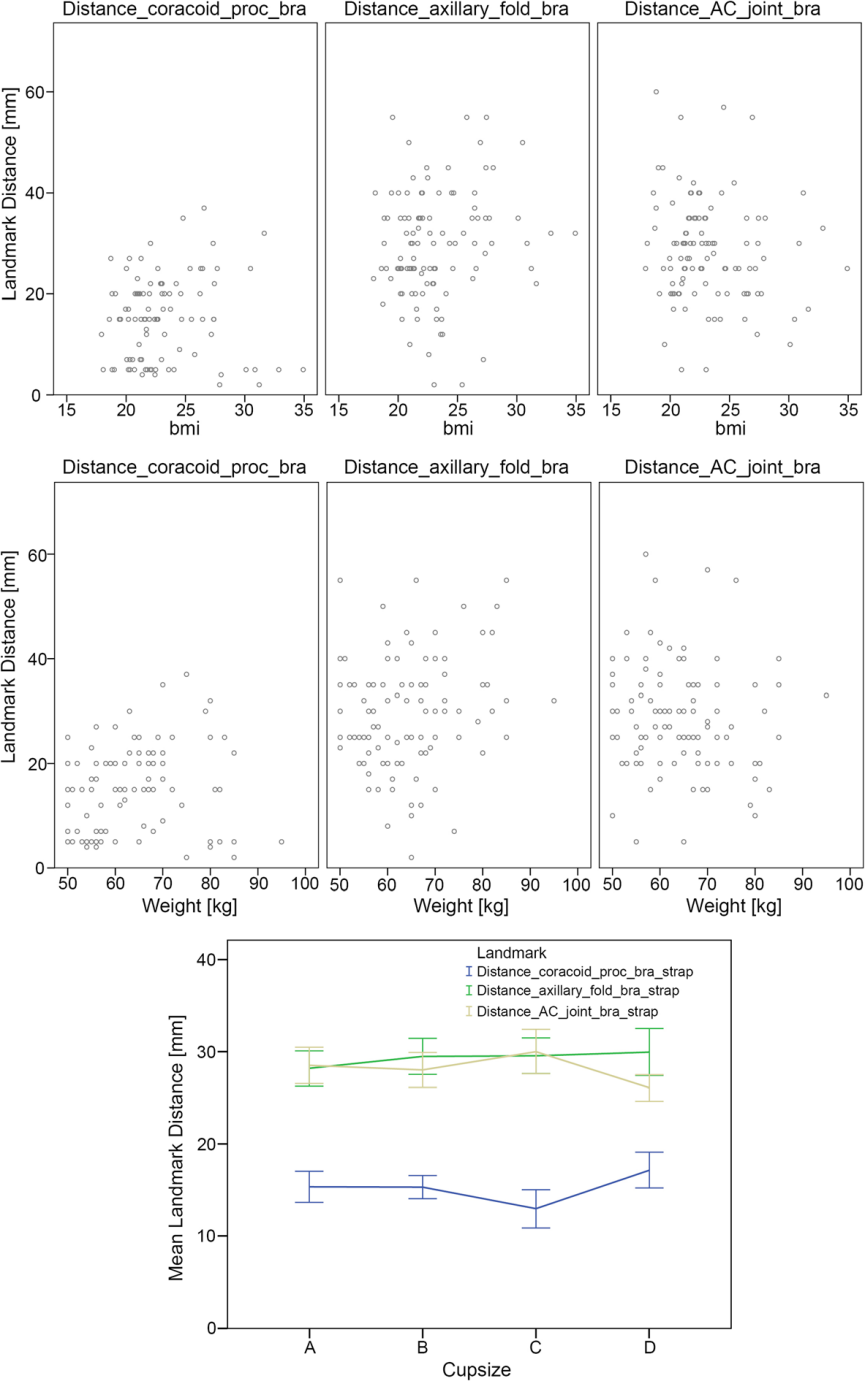
Relation between distance measurements and cup sizes

None of the measured distances revealed a significant linear relationship with any of the demographic variables (cup size, body weight, or BMI) in the ordinary least square linear regression analysis—see Fig. 4.

## Discussion

The fact that a coverable skin incision after shoulder surgery is desired by female patients but non-coverable skin incision is generally used in shoulder surgery led us to perform this study to meet the women’s demand of an optimal shoulder scar.

The bra strap incision is not a new incision. It might have been used by many surgeons for many years, but to our knowledge, it has never been published so far. The alteration of the bra strap incision compared to the standard skin incision for the deltopectoral approach is minimal and does not affect the clinical outcome after the Latarjet procedure. In this study, the scar was only used in stabilization procedures (Latarjet) but the coverable skin incision can also be used for surgical procedures on the clavicle, the AC joint, or shoulder joint.

The clinical results from this study are almost identical to the results seen in prior published data from our institution, in which 49 patients underwent a Latarjet procedure [17]; 12 of the 49 patients were female patients. For the purpose of this study, we analyzed the clinical results of the 12 female patients from this prior study to compare the results with the results from this study. The mean absolute CMS of 86 points (range, 40–100), the mean relative RCS of 92% (range, 83–100), and mean SSV of 79% (range 0–100) show no statistical differences to the clinical results from this study. A potential risk could be that the bra strap may irritate the subjacent scar tissue. However, the main pressure of the bra strap is seen at the clavicle or the acromioclavicular joint, which supports that all 18 patients denied scar pain in general or pain that would be elicited by bra strap pressure.

The bra strap incision can only be covered in bras which have a lateral strap, which runs vertically up towards the AC joint. These types of bras are by distant the most used and sold bras worldwide. Nevertheless, there are other types of bras available without straps or with triangular straps which are mounted with each other behind the neck. These types of bras would not allow coverage of the resulting bra strap incision scar.

Leslie et al. described an axillary incision for the deltopectoral approach [10] to gain a cosmetic advantage because the incision can be hidden in the axilla. Even though the axillary skin is easily moveable, the axillary incision is located too inferiorly to be suitable for the open Latarjet procedure as the coracoid process, which has to be addressed and mobilized, lies too superiorly.

The study only included female patients who underwent the open Latarjet procedure. Distal or midshaft clavicular fractures, coracoclavicular ligament reconstruction, tendon transfers like pectoralis major transfers, or shoulder arthroplasty are other interventions in which the bra strap incision might be useful.

The prediction of the bra strap course would be helpful for surgeons who want to meet the aesthetic demands of female patients. The elaborated mean distances from the three well palpable and visible shoulder anatomical landmarks to the longitudinal center of the bra strap allow an estimation of the general course of the bra strap. The tip of the coracoid process is within a mean lateral distance of 15 mm closer to the bra strap center than the more laterally placed acromioclavicular joint and axillary fold with a mean lateral distance of 28 and 30 mm, respectively.

Nevertheless, the mean distances showed high variability, which makes a reliable prediction of the bra strap course and therefore makes the drawing of the coverable bra strap incision difficult. Therefore, preoperative marking of the bra strap courses is still necessary if the surgeon desires to achieve the bra strap incision with its resulting scar.

There are two major limitations of the study. First, to confirm the safety of the altered incision technique, more patients would have been needed and should have been compared to a control group. Nevertheless, the clinical results from this study are almost identical with prior data with the use of an oblique incision [8, 17]. Second, thoracic landmarks were not evaluated because the main part of the thorax was covered by draping during shoulder surgery. With a change of shoulder draping technique toward a more medially exposed operating field, the thoracic landmarks might be visible and used for additional orientation of the bra strap skin incision for the deltopectoral approach. Therefore, the measurement of distances from the deltopectoral skin incision to medial thoracic landmarks might be of interest but was not addressed in this study.

## Conclusion

The bra strap incision for the open Latarjet procedure was welcomed by all patients included in this survey. The change of the incision orientation did not compromise technical feasibility and clinical results. Optimally, the skin incision is preoperatively marked in agreement with the patient. If this is not possible, the mean distances from the bra strap center to the acromioclavicular joint, the tip of the coracoid process, and the top of the anterior axillary fold lie approximately 28 mm, 15 mm, and 30 mm lateral to the bra strap center, respectively.

Nevertheless, the variability of these mean distances is too high to reliably predict the bra strap course, which makes preoperative marking of the bra strap course more favorable for the optimal coverable incision placement.

## Abbreviations

BMI: Body mass index
CMS: Constant and Murley score
SSV: Subjective shoulder value
